# Impact of 18F-FDG PET/CT on Clinical Management of Suspected Radio-Iodine Refractory Differentiated Thyroid Cancer (RAI-R-DTC)

**DOI:** 10.3390/diagnostics11081430

**Published:** 2021-08-07

**Authors:** Elisa Lodi Rizzini, Andrea Repaci, Elena Tabacchi, Lucia Zanoni, Valentina Vicennati, Ottavio Cavicchi, Uberto Pagotto, Alessio Giuseppe Morganti, Stefano Fanti, Fabio Monari

**Affiliations:** 1 Radiation Oncology Unit, IRCSS Azienda Ospedaliero-Universitaria di Bologna, 40138 Bologna, Italy; amorganti60@gmail.com (A.G.M.); fabio.monari@aosp.bo.it (F.M.); 2 Division of Endocrinology, IRCSS Azienda Ospedaliero-Universitaria di Bologna, 40138 Bologna, Italy; andrea.repaci@aosp.bo.it (A.R.); vicennati@aosp.bo.it (V.V.); uberto.pagotto@unibo.it (U.P.); 3 Nuclear Medicine Unit, IRCSS Azienda Ospedaliero-Universitaria di Bologna, 40138 Bologna, Italy; tabacchielena@libero.it (E.T.); lucia.zanoni@aosp.bo.it (L.Z.); stefano.fanti@aosp.bo.it (S.F.); 4 Department of Otolaryngology Head and Neck Surgery, IRCSS Azienda Ospedaliero-Universitaria di Bologna, 40138 Bologna, Italy; ottavio.cavicchi@aosp.bo.it; 5 Department of Experimental, Diagnostic and Specialty Medicine–DIMES, Alma Mater Studiorum, University of Bologna, 40138 Bologna, Italy

**Keywords:** 18F-FDG PET/CT, clinical management, radioiodine refractory, recurrent/persistent advanced DTC

## Abstract

Background: As reported in the literature, [18F]-fluorodeoxyglucose positron emission tomography/computed tomography ([18F]-FDG PET/CT) provides useful qualitative and semi-quantitative data for the prognosis of advanced differentiated thyroid cancer. Instead, there is a lack of data about the real clinical impact of 18F-FDG PET/CT on the choice of the more effective therapeutic approach for advanced differentiated thyroid cancer (DTC) that starts to lose iodine avidity. The primary aim of this retrospective study was to assess how 18F-FDG PET/CT can guide the choice of the best therapeutic approach to RAI-refractory DTC (RAI-R-DTC) in patients with a doubtful iodine uptake/negative 18F-FDG PET/CT I whole-body scan after several radioactive iodine therapies (RAIT). The secondary aim was to assess the prognostic role of clinical and semi-quantitative metabolic 18F-FDG PET/CT parameters in comparison to published data. Materials and methods: A monocentric retrospective observational study was performed, reviewing the medical records of 53 patients recruited from a database of 208 patients treated at our Institution between 2011 and 2019, with advanced DTC that underwent FDG PET/CT scan for a suspected RAI-R-DTC. Selected patients had to perform a 18F-FDG PET/CT scan after the second RAIT based on a doubtful iodine uptake/negative 131 I whole-body scan and/or persistent elevated thyroglobulin levels. Metabolic response was defined according to positron emission tomography response criteria in solid tumors (PERCIST) guidelines. Standardized uptake value (SUV)max, SUVmean, metabolic tumor volume (MTV), and total lesion glycolysis (TLG) were calculated. The association between metabolic features, clinical parameters and progression free survival (PFS) was assessed applying Kruskal–Wallis, chi-square-Pearson correlation tests, and Cox regression analyses when appropriate. Results: Among our sample of 53 patients (mean age 52.0 ± 19.9 years; 31 women and 22 men), 27 (51.0%) presented a positive 18F-FDG PET/CT scan: 16 (59.0%) underwent watchful waiting, 4 (15.0%) received external-beam radiation therapy (EBRT), 4 (15.0%) underwent surgery, 2 (7.4%) received another course of RAI therapy, and 1 underwent surgery + EBRT. PERCIST response was evaluated in 14/27 patients. Median follow-up was 5.8 ± 3.9 years and median PFS was 38.0 ± 21.8 months. At the last follow-up assessment, 14/53 (26.4%) demonstrated disease progression, 13/53 (24.5) persistence of structural disease, 25/53 (47%) persistence of biochemical disease, and 15/53 (28%) had an excellent response. A significant association was found between therapeutic approach, metabolic response, and final disease response evaluation, as well as a linear correlation between MTV and TLG with thyroglobulin level. Conclusions: Our Institutional experience confirmed the role of 18F-FDG PET/CT as a useful guide in the clinical management of RAI-R-DTC and obviated further unnecessary RAIT.

## 1. Introduction

Though classified as a rare tumor, thyroid cancer is the most common endocrine malignancy and its incidence has been increasing worldwide. In most cases, thyroid cancer is a non-aggressive tumor and it is treated effectively with standard primary treatments. In 5–10% of patients, however, advanced disease carries a higher probability of local recurrence or metastatic disease. Metastasis is the main cause of thyroid cancer-related deaths. The overall mortality rate depends on individual factors [1] and ranges between 65% and 75% at 5 and 10 years, respectively, in disseminated metastatic disease [2,3,4]. Two thirds of patients present with iodine-sensitive disease, for which RAIT is considered the gold standard, and prognosis remains favorable. The remaining one third, however, present with RAI-R-DTC in which neoplastic lesions have a low avidity for iodine and prognosis varies for this reason [4]. Early identification and a multidisciplinary approach to this patient subgroup are crucial to obviate unnecessary treatment such as RAIT, which may pose adverse/toxic effects of high cumulative 131I activity and delay the beginning of other potentially more effective therapies.

18F-FDG PET/CT has become an important tool in the postoperative management of patients with DTC, in disease staging and the follow-up of high-risk patients. Its diagnostic accuracy is highest in the evaluation of patients with “thyroglobulin elevated but negative iodine scintigraphy Syndrome” (TENIS syndrome) and in the early identification of RAI-R-DTC [5,6]. Empiric RAIT was demonstrated ineffective in patients with RAI-R-DTC, whereas 18F-FDG PET/CT was shown to reduce the number of ineffective RAIT and to provide guidance for loco-regional therapy such as surgery or external-beam radiation therapy (EBRT) [7,8]. However, published data are scarce about the real impact of 18F-FDG PET/CT on treatment decision making for this subgroup of patients with disease persistence who do not respond to RAIT. The primary aim of this retrospective study was to assess the impact of 18F-FDG PET/CT on the choice of the best therapeutic approach to RAI-R-DTC after the second RAIT. The secondary aim was to assess the prognostic role of clinical and semi-quantitative metabolic 18F-FDG PET/CT parameters in comparison to published data.

## 2. Materials and Methods

For this retrospective, single center study, from a database of 208 patients affected by persistent/recurrent DTC treated between January 2011 to July 2019 at the Radiation Oncology Center, Sant’Orsola-Malpighi Hospital of Bologna, we selected 53 patients according to the inclusion criteria as follows: at least two previous RAIT and a 18F-FDG PET/CT scan performed after the second RAIT because of clinical suspicion of RAI-refractory disease supported by a doubtful/negative iodine scintigraphy and/or elevated levels of suppressed/stimulated thyroglobulin. Routine clinical follow-up included annual thyroglobulin level testing and a PET/CT scan or a CT scan when disease progression was suspected, assessed according to PERCIST [9] and RECIST 1.1 criteria, respectively [10].

For the analysis we subdivided the sample into four groups according to positive or negative findings on 18F-FDG PET/CT and post-treatment 131I whole body scintigraphy (Rx-WBS) after the second RAIT (131I/FDG groups).

18F-FDG PET/CT images were acquired using a hybrid PET/CT scanner according to standard EANM guidelines [11]; PET/CT scans were acquired on a 3D tomograph (Discovery STE; General Electric Healthcare, Milwaukee, WI, USA). Images were retrospectively reviewed (Advantage 4.5 version workstations, GENERAL ELECTRIC COMPANY, Italy) by two nuclear medicine physicians (E.T. and E.L.R.). All scans were acquired during hormonal replacement therapy. Semi-quantitative analysis was performed using PET Volume Computer-Assisted Reading software (VCAR, GE Healthcare, Italy) to measure metabolic parameters: maximum and mean standardized uptake values (SUVmax and SUVmean), total metabolic tumor volume (MTV), and total lesion glycolysis (TLG). Based on published data [12], we subdivided the SUVmax into three thresholds (<5, 5–10, and >10). For the patients with FDG-avid lesions we evaluated disease response by calculating the peak of standardized uptake value normalized for lean body mass (SULpeak) according to PERCIST criteria.

For all patients we collected and analyzed the data on disease response to the therapeutic PET/CT guided approach of persistence/recurrence DTC after the second RAIT (first restaging) and the disease response at the latest follow-up (final restaging), both of which were classified according to the 2015ATA guidelines [1].

Progression free survival (PFS) was defined as the period between the starting date of the first therapeutic PET/CT guided approach after the second RAIT and the date of progressive disease for PET-positive patients and the period between the second RAIT and the date of progressive disease for PET-negative patients. Generally, we considered surgery when target had a significant FDG uptake (SUVmax > 5) and at the same time surgery was feasible, conversely patient underwent EBRT when was not surgery candidate (comorbidities, age, target dimensions, etc.). We have chosen observation when FDG uptake was under the SUVmax cut-offs and/or Tg levels were substantially stable. For the analysis, we subdivided PFS in three periods (<12 months, 12–24 months, and >24 months). The surviving patients who were progression free were censored at the date of the latest follow-up.

To evaluate the impact of 18F-FDG PET/CT on decision making, we searched for an association between the variables therapeutic PET/CT guided approach for the persistent/recurrent disease and first restaging data and between therapeutic PET/CT guided approach and PERCIST response. Additionally, we analyzed possible associations between PET positive/negative groups combined with first restaging and final restaging and between therapeutic PET/CT guided approach combined with first restaging and final restaging. Furthermore, to evaluate the second endpoint, we explored the relationship between the metabolic parameters (MTV, TLG, SUVmax and SUVmean) of the PET/CT scan after the second RAIT and PFS and disease response at final restaging. Analysis of correlations between metabolic 18F-FDG PET/CT parameters and thyroglobulin levels after the first and the second RAIT was also performed.

Written, informed consent for data collection for scientific purpose was collected from all patients. The study was approved by the Sant’Orsola-Malpighi Hospital Ethics Committee (0008743-ENT COBRA Prot.-standardized data collection for patients treated with metabolic and external beam radiotherapy).

### Statistical Analysis

Continuous variables are expressed as the mean, median, mode, and standard deviation. Non parametrical test (Kruskal–Wallis test) was used to compare the distribution of the means. Associations between groups were analyzed using Pearson’s chi-squared test. Cox regression multivariate analysis was used to estimate relationships between the clinical data, the metabolic parameters, and the final restaging data. Kaplan-Meyer curves were used to evaluate the risk of disease persistence in relation to lesions with increased FDG uptake. All analyses were performed with SPSS Statistics (IBM Corp. Released 2011. IBM SPSS Statistics for Windows, Version 20.0. Armonk, NY, USA).

## 3. Results

Our study included 53 patients in the final analysis (mean age at diagnosis, 52 ± 19.9 years; 31 women and 22 men). The clinical, histopathological, and laboratory data are shown in Table 1. According to 2004 World Health Organization (WHO) classification, 73.5% of the patients had a non-aggressive DTC histological type and 26.5% an aggressive tumor (Hurtle cell, tall cell, solid/trabecular variants, as it shows in Table 1A). All the patients underwent total thyroidectomy; prophylactic neck dissection (central or unilateral lymph node dissection) was performed at the discretion of endocrine surgeon.

After the first RAIT, 20/53 patients had already undergone the first 18F-FDG PET/CT scan, which was already positive in 8/20. Before the second RAIT, 9/53 (17%) patients underwent surgery, 4/53 (7.5%) EBRT, and 1/53 (1.9%) both surgery and EBRT. Only observation was done for 36/53 (67.9%) patients and 3/53 (5.7) patients only underwent to a biopsy to investigate the FDG-PET uptake.

Table 2 presents the PET/CT imaging findings after the second RAIT; the same lesions were detected on both the PET/CT and the Rx-WBS scan in 20/53 (37.7%); lesions differed between PET/CT and Rx-WBS scans in 9/53 (15%); lesion number and site differed between the PET/CT and the Rx-WBS scans in 24/53 (45%).

Therapeutic approach guided by positive 18-FDG-PET/CT scan was surgery for 4/27 (15%) patients, EBRT for 4/27 (15%) patients, another RAIT for 2/27 (7.4%) patients, combined surgery and EBRT for 1/27 (1.9%) patient. Only observation was done for 16/27 (59%) patients.

The median follow-up since initial diagnosis was 5.8 ± 3.9 years (range, 2.3–20.3). PERCIST response was evaluated in 14/27 patients: complete metabolic response was recorded in 7/27, stable metabolic disease in 2/27, partial metabolic response in 1/27, and progressive metabolic disease in 4/27. During follow-up, based on RECIST/PERCIST criteria, disease progression was recorded in 14/53 (26.4%) patients. The median PFS was 38.0 ± 21.8 months. At the last follow-up, 47/53 (88.7%) patients were alive while 2/53 (3.7%) had died for disease progression (Table 2).

Metabolic parameters analyzed were: mean MTV 18.8 ± 60.5 cm^3^, mean TLG 35 ± 81.3, mean SUVmax 7.9 ± 6.9 (range, 1.5–36.3), mean SUVmean was 3.3 ± 2.4 and mean SULpeak was 4.2 ± 3.4 g/mL (range, 0.8–18.5).

Distribution of the means for the 131I WBS/FDG groups and the main variables related to the treatments are presented in Table 3; there was a statistically significant difference between basal and stimulated thyroglobulin levels before the first RAIT (*p* = 0.013, *p* = 0.004), stimulated thyroglobulin before the second RAIT (*p* = 0.028), and the 131I cumulative dose at the second RAIT (*p* = 0.020).

Analysis of the association between the variables and the 131I WBS/FDG groups showed a statistical significance for N and M (*p* = 0.039, *p* = 0.002) and final restaging data (*p* = 0.004) (Table 4). Analysis showed that the majority of patients with negative Rx-WBS scan and positive 18F-FDG PET/CT scan had structural disease with persistence/recurrence of disease at last follow-up (Table 3). Conversely, no significant associations were found between the131I WBS/FDG groups and SUVmax range (*p* = 0.833). Though no statistical significance was found, our data indicated that low FDG uptake can also characterize disease with high-risk features, as noted for the patients with a faint positive PET/CT scan.

Regarding our first study endpoint, we found a statistically significant association between the type of therapeutic approach guided by FDG-PET/CT scan after the second RAIT and the first restaging data (*p* = 0.011) (Table 5); particularly patients who underwent surgery and/or EBRT guided by PET/CT imaging had more excellent response or biochemical persistence of disease without structural progression than the others. No significant association between therapeutic PET/CT guided approach and PERCIST response was found. Table 6 also shows possible associations of three variables contemporaneously to find a possible predictive relationship between type of therapeutic approach guided by PET/CT and disease response. We found a statistically significant association between therapeutic PET/CT guided approach and first and final restaging, particularly in patients in which excellent response (*p* = 0.026) or biochemical persistence of disease (*p* = 0.013) at final restaging was recorded: about 50% of patients with persistence of biochemical or structural disease at first restaging after surgery and/or EBRT were disease free at the last follow-up assessment.

About our second endpoint, no significant difference between the distribution of PFS range and metabolic parameters or between metabolic parameters and restaging data was found (Table 7). However, there was a significant inverse association between PFS and PERCIST response (*p* = 0.02) and PFS and final restaging data (*p* = 0.05); PFS was longer in patients with a better disease response, while no association was found between lesion uptake (SUVmax) and PFS (*p* = 0.740) (Table 4).

**Figure 1:**
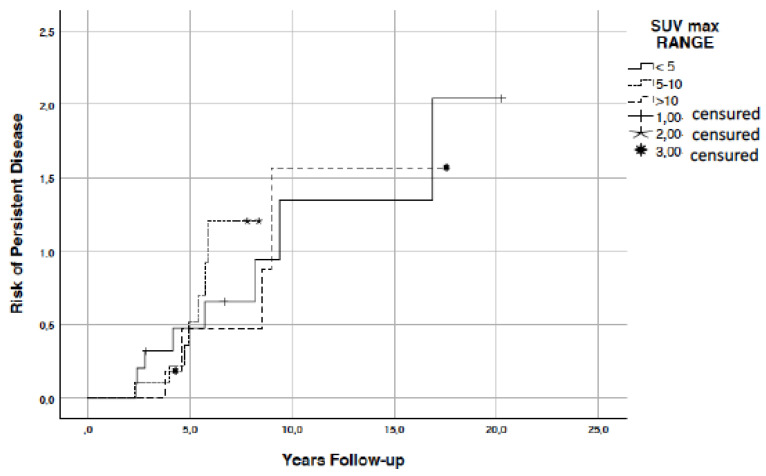
Kaplan-Meyer curves.

**Figure 2:**
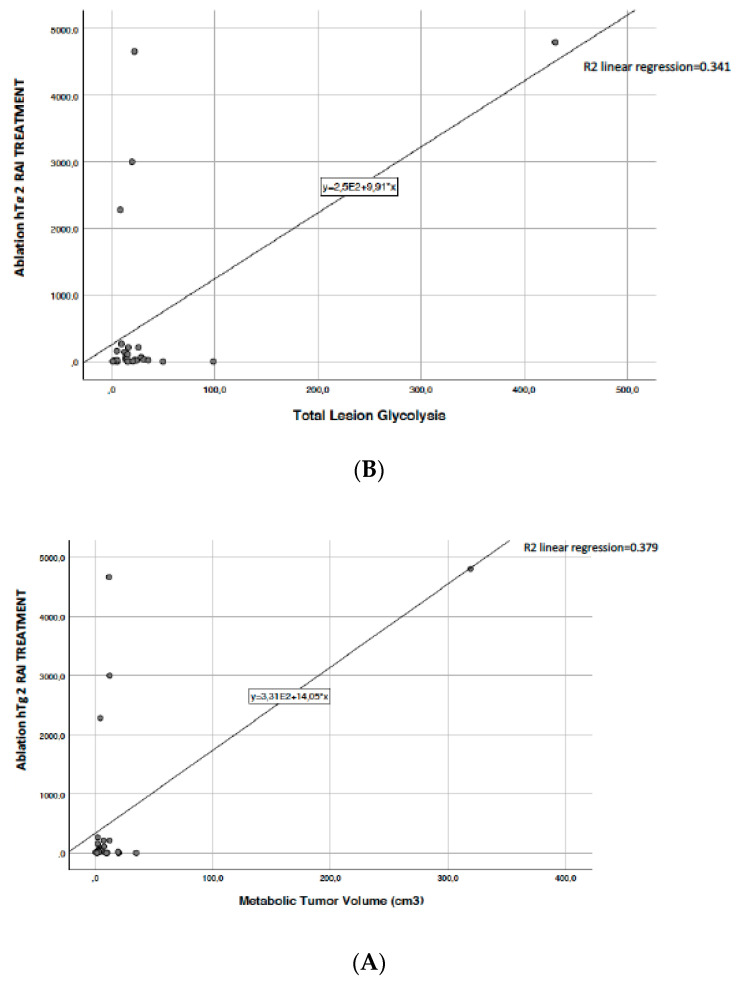
Correlation between MTV (Panel **A**), TLG (Panel **B**), and stimulated thyroglobulin.

Kaplan-Meyer curves (Figure 1) showed that the final outcome is not influenced by the SUVmax range of hypermetabolic lesions. Differently, we found a significant correlation between MTV, TLG, and stimulated thyroglobulin (Figure 2A,B) (*p* = 0.001).

Cox regression multivariate analysis showed that MTV *p* = 0.034, hazard ratio (HR) 1.100, 95% confidence interval (CI 1.007–1.202) and PERCIST response (*p* = 0.004, HR 2.434, 95% CI 1.322–4.481) were associated independently with persistent disease.

## 4. Discussion

Distant metastases develop in about 10% of patients with DTC. RAIT is curative in only about one third of such patients because the tumor may present initially as non-iodine-avid disease or gradually lose the ability to concentrate iodine due to impaired iodine-metabolizing gene expression. Although controversial, major guidelines still suggest repeated RAIT until the standard cumulative dose of 600 mCi is reached in cases of persistence/recurrence of metastatic disease [1,13]. Many retrospective studies have found 18F-FDG-PET/CT to be a valid, prognostic tool in patients with elevated thyroglobulin but negative ultrasound neck and 131I WBS [14]. Dong et al. reported a pooled specificity and sensitivity of 93.5% and 84.7%, respectively [15], with the limits of the detection system, manifold patient variables (thyroglobulin cut off, clinical characteristics, histological tumor types), and TSH stimulation test. Deandreis et al. demonstrated that FDG uptake has a highly prognostic value for survival because FDG avidity and number of FDG-avid lesions are signs of aggressive disease [16]. In 2000, Wang et al. noted that FDG volume >125 mL was associated with worse survival [17]. More recently, prognostic performance is evaluated by quantitative analysis of metabolic parameters (e.g., SUVmax, SUVmean, SULpeak, MTV, and TLG) [18]. Manohar et al. demonstrated that MTV and TLG correlated with thyroglobulin doubling-time and are significantly associated with OS and PFS and that they could be used for dynamic risk stratification of patients with metastatic RAI-refractory DTC [19].

Several studies have described the use of PET/CT imaging in the staging and the follow-up of high-risk patients [20]. A study by Pomerri et al. reported a positive impact of 18F-FDG PET/CT on the detection of resectable tumor recurrence and additional tumor sites, but the follow-up period was too short [21]. Giovanella et al. found that PET/CT findings change patient management in 20–40% of cases [22]. In their review study, Ciarallo et al. reported that 18F-FDG PET/CT changes the course of management in 14% to 78% of patients with suspected disease recurrence [23].

In the present retrospective study, the small size and inhomogeneity of the sample preclude definition of a significant percentage of the real impact of 18F-FDG PET/CT in patients with persistent/recurrent disease. What our findings do show is that PET-CT was crucial in selecting the most suitable therapeutic approach to persistent/recurrent disease, in detecting radioactive-iodine refractoriness of the tumor before reaching the conventional cumulative 131I dose of 600 mCi, ultimately obviating ineffective and toxic RAIT. Patients of our sample underwent only two RAI course at the time of PET imaging but the features of their disease (negative or doubting Rx-WBS scan, aggressive histological type, persistence of elevated serum tg levels) led us to define them as iodiorefractory. Of relevance, in our opinion, the fact that only 2/27 patients with a positive PET/CT scan underwent further RAI courses and both had persistence of structural disease at final restaging was relevant; the other patients did not undergo further and probably unnecessary RAIT; PET scan guided the choice of the alternative undertaken therapies. Furthermore, 11/27 (41%) patients with a positive PET/CT scan and 3/26 (11.5%) with a negative PET/CT scan had persistence of structural disease at first restaging. There were no statistically significant differences in outcome between the two groups at final restaging because of effective local control. Moreover, the patients who underwent EBRT had good loco-regional control at the first restaging with no local progression of disease at the last follow-up.

Despite the small study sample, we may also state that the patients who received local therapy (surgery and/or EBRT) after the second RAIT achieved excellent response.

About the prognostic role of [18F]-FDG PET/CT, we searched for a possible association among the metabolic parameters with the main variables of tumor aggressiveness and published data. We found a significant correlation between MTV and TLG and thyroglobulin levels and final outcome data (R^2^ = 0.341, Figure 2 panel B). MTV was an independent factor associated with high probability of disease persistence (R^2^ = 0.379, Figure 2 panel A). Our analysis confirmed the association between the presence of a hypermetabolic lesion and the concomitant absence of 131I uptake. Patients with high metabolic tumor burden and a negative Rx-WBS scan also had higher thyroglobulin levels and a poorer outcome at final restaging. Conversely, we found no significant association between SUVmax and 131I/FDG groups, SUVmax and PERCIST response, SUVmax and final outcome. Nevertheless, these findings should not discourage the routine use of 18F-FDG PET/CT but rather should alert attention to the low uptake of lesions because low SUVmax is not always a sign of favorable prognosis.

A few study’s limitations should be considered, for instance, the retrospective nature; furthermore, the extreme heterogeneity and variability of patient-dependent factors and of the natural history of thyroid neoplasms. Moreover, the small sample size dictated by the need for longer follow-up and the absence of a second PET/CT scan for the PERCIST evaluation created a statistical bias.

Further studies are needed to generate prognostication algorithms for the use of metabolic parameters. Randomized trials are needed to confirm the real impact of 18F-FDG PET/CT on the clinical management of RAI-refractory disease.

Our experience suggests that the use of 18F-FDG PET/CT led to a significant reduction in the blinded RAI administration, which is ineffective in metastatic non-iodine-avid thyroid disease. Finally, patients with hypermetabolic lesions could benefit from the integration of multiple therapies for better disease control and QoL improvement.

## Figures and Tables

**Table 1:** Patient characteristics and clinical history.

		No of Patients	%
**SEX**	FEMALE MALE	31 22	58.5 41.5
**HISTOLOGY**	CLASSICALPAPILLARY FOLLICULAR PAPILLARY FOLLICULAR WIDELY INVASIVE HURTLE CARCINOMA PAPILLARY TALL CELL VAR. PAPILLARY MIXED VAR. INSULAR PAPILLARY DIFFUSE SCLEROSANT PAPILLARY SOLID VARIANT PAPILLARY COLUMNAR CELL VAR FOLLICULAR MIN INVASIVE	30 8 4 2 2 2 1 1 1 1 1	56.6 15.1 7.5 3.8 3.8 3.8 1.9 1.9 1.9 1.9 1.9
**Stage T at diagnosis (AJCC7)**	pT1a pT1b pT2 pT3 pT4a pT4b	6 5 6 27 6 3	11.3 9.4 11.3 50.9 11.3 5.7
**Stage N at diagnosis (AJCC7)**	pN0 pN1a pN1b pNx	9 18 14 12	17.0 34.0 26.4 22.6
**Stage M at diagnosis (AJCC7)**	M0 M1	49 4	92.5 7.5
**LVI**	Yes No	21 32	39.6 60.4
**EC**	Yes No	6 47	11.3 88.7
**R1**	Yes No	20 33	37.7 62.3
**R2**	Yes No	4 49	7.5 92.5
**Risk Stratification** **(ATA 2015)**	LOW LOW/INTERMEDIATE INTERMEDIATE INTERMEDIATE/HIGH HIGH	7 4 11 15 16	13.2 7.5 20.8 28.3 30.2
**THYROGEN use at the first RAIT**	Yes No UNKNOWN	28 24 1	45.3 52.8 1.9
**Rx-WBS first course of RAI**	NEGATIVE RESIDUAL UPTAKE LYMPH NODE UPTAKE DISTANT LESION UPTAKE	2 42 3 6	3.8 77.4 5.7 11.3
**THERAPEUTIC APPROACH BEFORE second RAIT**	OBSERVATION SURGERY RTE BIOPSY SURGERY + EBRT	36 9 4 3 1	67.9 17.0 7.5 5.7 1.9
**THYROGEN use at the second RAIT**	Yes No	8 45	15.1 84.9
**Rx-WBS at second RAIT**	NEGATIVE RESIDUAL UPTAKE LYMPH NODE UPTAKE DISTANT LESION UPTAKE	30 14 2 7	56.6 24.6 3.8 13.2

LVI: lymphovascular invasion; EC: extracapsular lymph node infiltration; Rx-WBS: post-therapy whole-body scan; RAI: radioactive iodine; EBRT: external beam radiation therapy, RTE real-time elastography, AJCC7 7th edition of the American Joint Commission on Cancer staging system; ATA 2015 American Thyroid Association Management Guidelines for Adult Patients with Thyroid Nodules and Differentiated Thyroid Cancer.

**Table 2:** Findings on Rx-WBS scan, 18F-FDG/PET scan, restaging, PERCIST response, and final outcome.

		No of Patients	%
**131I-FDG**	131I−/FDG− 131I+/FDG− 131I+/FDG+ 131I−/FDG+	24 2 7 20	45.3 3.8 13.2 37.7
**Hypermetabolic** **Lesion**	NONE LYMPH NODES BONE LUNG MULTIPLE SITES	26 15 3 3 6	49.1 28.3 5.7 5.7 11.3
**SUVmax range**	<5 5–10 >10	11 10 6	20.8 18.9 11.3
**SUVmean**		27	50.9
**MTV**		27	50.9
**TLG**		27	50.9
**SULpeak**		27	50.9
**PERCIST RESPONSE**	Not available CMR SMD PMR PMD	13/27 7/27 2/27 1/27 4/27	48.1 25.9 7.4 3.7 14.8
**Therapeutic FDG PET/CT guided approach**	OBSERVATION SURGERY EBRT RAI SURGERY + EBRT	16/27 4/27 4/27 2/27 1/27	59.2 14.8 14.8 7.4 3.7
**FIRST RESTAGING**	EXCELLENT RESPONSE BIOCHEMICAL PERSISTENCE STRUCTURAL PERSISTENCE	4 34 15	7.5 64.0 28.0
**FINAL RESTAGING**	EXCELLENT RESPONSE BIOCHEMICAL PERSISTENCE STRUCTURAL PERSISTENCE	15 25 13	28.3 42.7 24.5
**PFS range (months)**	<12 12–24 >12	8 11 34	15.1 20.8 64.2
**OS**	ALIVE DEATH DISEASE RELATED DEATH NON DISEASE RELATED	47 2 4	88.7 3.7 7.5

SUV: standardized uptake value; MTV: metabolic total volume; TLG: total lesion glycolysis; SULpeak: peak of standardized uptake corrected for lean body mass; PFS: progression free survival; OS: overall survival; CMR: complete metabolic response; SMD: stable metabolic disease; PMR: partial metabolic response; PMD: progression metabolic disease; EBRT: external beam radiation therapy.

**Table 3:** Distribution of the means of the 131I/FDG subgroups and related variables (Kruskal–Wallis test).

		131I−/FDG−	131I+/FDG−	131I+/FDG+	131I−/FDG+	*p* Value
**Age**	**1RAI**	49.7 ± 14.6	63.7 ± 13.3	63.8 ± 16.4	49.8 ± 25.5	0.187
	**2RAI**	51.2 ± 14.4	63.5 ± 13.4	65.5 ± 16.2	58.4 ± 20.8	0.127
**Basal TG**	**1RAI**	14.6 ± 42.7	1512.8 ± 1994	1646.2 ± 2014.0	4.7 ± 8.4	**0.013**
	**2RAI**	3.5 ± 4.8	160.0 ± 217.7	950.0 ± 1816.0	38.9 ± 100.5	0.234
**Stimulated** **TG**	**1RAI**	47.3 ± 83.9	3000.0 ± 0.0	1830.0 ± 1809.0	109.4 ± 319.2	**0.004**
	**2RAI**	19.2 ± 22.1	968.6 ± 1305	1459.0 ± 1929.0	295 ± 1030	**0.028**
**DeltastimhTG**		53.9 ± 259.8	−67.7 ± 43.5	−26.4 ± 77.9	727.9 ± 2585	0.334
**Cumulative 131I dose**		232.9 ± 52.8	297.9 ± 1.8	313.7 ± 74.2	227.2 ± 57.9	**0.020**
**MTV**				50.7 ± 118.4	3.5 ± 1.7	0.361
**TLG**				78.3 ± 155.4	19.8 ± 22.3	0.213
**SUVmax**				10.8 ± 12.0	6.9 ± 4.0	0.825
**SUVmean**				4.4 ± 4.3	2.9 ± 1.3	0.618
**SULpeak**				6.1 ± 6.1	3.5 ± 1.7	0.912
**Follow up (years)**		6.2 ± 2.3	5.3 ± 1.0	5.2 ± 5.5	8.0 ± 4.9	0.076
**PFS (months)**		42.5 ± 21.3	53.5 ± 14.8	21.2 ± 16.4	36.0 ± 22.2	0.079

SD denotes Standard Deviation, SUV standardized uptake value, MTV metabolic total volume, TLG total lesion glycolysis, SULpeak peak of standardized uptake value corrected for lean body mass, PFS progression free survival, TG thyroglobulin, RAI radioactive iodine, plus-minus values are the means ± SD.

**Table 4:** Associations between variables.

VARIABLES		*p* Value
**131I/FDG groups**	SEX	0.600
	HISTOLOGY	0.127
	T	0.107
	N	**0.039**
	M	**0.002**
	LVI	0.502
	EC	0.097
	R1	0.597
	R2	0.114
	RISK	0.075
	SUVmax range	0.833
	First restaging	**0.004**
	Final restaging	0.098
	PFS range	0.364
**SUVmax** **range**	PET+/− GROUPS	0.833
	Hypermetabolic lesion site	0.109
	PERCIST response	0.714
	Final restaging	0.954
	PFS range	0.740
**PFS range**	PET+/− GROUPS	0.149
	Hypermetabolic lesion site	0.693
	PERCIST response	**0.020**
	Final restaging	**0.050**
**Therapeutical PET/CT guided approach**	PERCIST response	0.091
	First restaging	**0.011**
	First and Final restaging	**0.012**
**FDG+/** **− groups**	First and Final restaging	0.070

**Table 5:** Association between treatment for persistence/recurrence of disease guided by PET/CT imaging and first restaging data.

		First Restaging		
		Excellent Response	Biochemical Persistence	Structural Persistence
**Persistence/recurrence treatment**	Observation	2.8%	77.8%	19.4%
	Surgery	40%	0.0%	60%
	EBRT	20%	60%	20%
	RAI	0.0%	0.0%	100%
	Surgery + EBRT	0.0%	100%	0.0%

EBRT: external beam radiation therapy; RAI: radioiodine.

**Table 6:** Association between 131I/FDG findings and N, M, and first restaging data.

		131I−/FDG−	131I+/FDG−	131I+/FDG+	131I−/FDG+
**N**	pN0	22.2%	0.0	0.0	77.8%
	pN1a	38.9%	11.1%	16.7%	33.3%
	pN1b	42.9%	0.0	28.6%	28.6%
	Nx	75%	0.0	0.0	25%
**M**	Mx	49%	4.1%	8.2%	38.8%
	M1	0.0%	0.0%	75.0%	25%
**First restaging**	Excellent response	100%	0.0%	0.0%	0.0%
	Biochemical persistence	64.5%	6.5%	3.2%	25.8%
	Structural persistence	14.3%	0.0%	28.6%	57.1%

**Table 7:** Association between PFS range, PERCIST response, and final restaging data.

		PFS <12 Months	PFS 12–24 Months	PFS >24 Months
**PERCIST response**	NA	40.0%	25.0%	64.3%
	CMR	0.0%	37.5%	28.6%
	SMD	0.0%	25.0%	0.0%
	PMR	0.0%	0.0%	7.1%
	PMD	60%	12.5%	0.0%
**Final restaging**	Excellent response	25%	9.1%	35.3%
	Biochemical persistence	25%	45.5%	52.9%
	Structural persistence	50%	45.5%	11.8%

NA: not available; CMR: complete metabolic response; SMD: stable metabolic disease; PMR: partial metabolic response; PMD: progressive metabolic disease; PFS progression free survival.
